# *In vitro* generation and bioactivity evaluation of C-reactive protein intermediate

**DOI:** 10.1371/journal.pone.0198375

**Published:** 2018-05-31

**Authors:** Jian-Min Lv, Ming-Yu Wang

**Affiliations:** MOE Key Laboratory of Cell Activities and Stress Adaptations, School of Life Sciences, Lanzhou University, Lanzhou, P.R. China; Consejo Superior de Investigaciones Cientificas, SPAIN

## Abstract

The conformational conversion of pentameric C-reactive protein (pCRP) to monomeric CRP (mCRP) has been shown to play important roles in the action of CRP in inflammation regulation. *In vivo* studies revealed the origin of mCRP and provided insights into how pCRP dissociation affected its functions. However, the interplay and exact bioactivities of CRP isoforms still remain uncertain due to the rapid conformational conversion and complex milieu *in vivo*. Herein, we have used surface-immobilization of pCRP to generate a preservable intermediate with dual antigenicity expression of both pCRP and mCRP. The intermediate has been further shown to exhibit modified bioactivities, such as a high affinity with solution-phase pCRP and an enhanced capacity of complement interaction. These results thus not only provide the conformational conversion details of CRP, but also propose a simple way *in vitro* to study how the functions of CRP are tuned by distinct isoforms.

## Introduction

C-reactive protein (CRP) is a pentameric protein playing important roles in inflammation in the human body[1, 2]. CRP has two naturally occurring and conformationally distinct isoforms, i.e., pentameric CRP (pCRP) and monomer CRP (mCRP)[3–5]. pCRP undergoes the conversion to mCRP under certain conditions. This process mainly involves disassembly of pentamer and epitope remolding of native subunit structure. Therefore, mCRP is different from native subunit in pentamer. Recent studies revealed that biological function of CRP mainly involves its conformation changes, and mCRP was indicated to be more active in exerting biological effects[6–10]. Moreover, the inter-subunit disulfide bond of CRP was also proved important to its conformation and activities[11, 12]. However, pCRP is very stable in the presence of calcium[13, 14] and its dissociation occurs mainly in denaturation conditions[1, 3, 13, 14].

Recently, several nondenaturing conditions have been proved to induce the dissociation of pCRP[8, 15, 16], among which our group identified a membrane-induced intermediate termed mCRP_m_[8]. The intermediate was shown to be dissociated CRP subunit with partly retained native conformation, suggesting the conversion of pCRP to mCRP and providing further insights into the functional mechanism of CRP *in vivo*[8, 15]. However, the generation of membrane-induced intermediate involves complex experimental steps and is time-consuming. Moreover, the prepared intermediate is difficult to be preserved.

Here we propose a new method to obtain the CRP intermediate in a simpler way. We show that immobilization of pCRP onto microtiter well surfaces can also induce conformational conversions and generate a CRP intermediate with both pentamer and monomer antigenicity expression. Furthermore, upon this relatively stable intermediate, a series of function evaluations could be performed easily but reliably, which may provide further insights into biological roles of CRP.

## Materials and methods

### Reagents

Human native CRP (purity>99%) was purchased from the Binding Site (Birmingham, UK). mCRP was prepared with urea-chelation treatment as before[3]. Mouse anti-human mCRP monoclonal antibody (mAb) (3H12, 8C10) and pCRP mAb (8D8, 1D6) were generated as described[17]. Sheep anti-human CRP polyclonal antibody (pAb) was purchased from the Binding Site.

PC-BSA (2 mol PC/mol BSA) was obtained from Biosearch Technologies (Novato, CA, USA). C1q was purchased from Abcam. Goat pAb against human C1q was from Calbiochem and HRP-conjugated rabbit anti-goat antibody was from Sigma-Aldrich (St. Louis, MO). Sheep anti-human C3d pAb and HRP conjugated donkey anti-sheep Ab were from the Binding Site and Sigma-Aldrich, respectively. Other reagents were from Sigma-Aldrich unless otherwise stated.

### Detection of pCRP and mCRP antigenicity expression

The antigenicity expression of pCRP and mCRP were determined with ELISA using conformational-specific mAbs. Briefly, after short washes, the wells with CRP immobilized were blocked with 1% BSA (unless stated otherwise to omit). Appropriate mAbs and HRP-conjugated secondary antibody incubations were performed in sequence. After antibody incubations, wells were briefly washed and developed with TMB and stopped with 1 M H_2_SO_4_. All the incubation steps in ELISA assay were conducted at 37°C, unless specified. The absorbance values at 450 nm were finally detected using a Varioskan Flash.

### Evaluation of complement interaction capacity

C1q binding capacity was evaluated by ELISA. Briefly, indicated concentrations of C1q was added to immobilized or bound proteins for 1 h after the microtiter wells were blocked with 1% BSA. After brief washes, a goat pAb against human C1q and HRP-conjugated rabbit anti-goat antibody were added successively, followed by TMB development, H_2_SO_4_ stop and absorbance values detection at 450 nm. For C3d deposition, microtiter wells with proteins immobilized were blocked with 1% BSA. 1% normal human serum (NHS) diluted in VBS^Ca+Mg^ (veranol buffered saline, VBS, pH 7.4, + 0.15 mM calcium and 0.5 mM magnesium), VBS^Mg+EGTA^ (VBS + 0.5 mM magnesium and 5 mM EGTA) or VBS^EDTA^ (VBS + 5 mM EDTA) with 1% BSA were added at 37°C for 30 min. After serum incubation and brief wash, sheep anti-human C3d pAb and HRP conjugated donkey anti-sheep Ab was used to detect the C3d deposition capacity. The following steps were conventional as before.

### Electron microscope (EM)

pCRP was firstly diluted into 25 μg/ml in 10 mM Tris and 140 mM NaCl. A 4μl droplet of protein was added to a freshly glow-discharged carbon coated 300 mesh copper EM grid for 10 s, followed by staining with uranyl acetate solution (1%, w/v) for 30 s. The grids were observed in a Tecnai G2 200 kV EM.

### Statistical analysis

Data were obtained from at least three independent experiments and represented as mean ± SEM. Data analysis was performed by one-way ANOVA using OriginPro 8 software (OriginLab Cooperation). A value of *P* <0.05 was considered significant. All data charts were also made using OriginPro 8 software.

## Results

### Surface immobilization of pCRP generated a conformational intermediate exhibiting dual antigenicity

**Fig 1:**
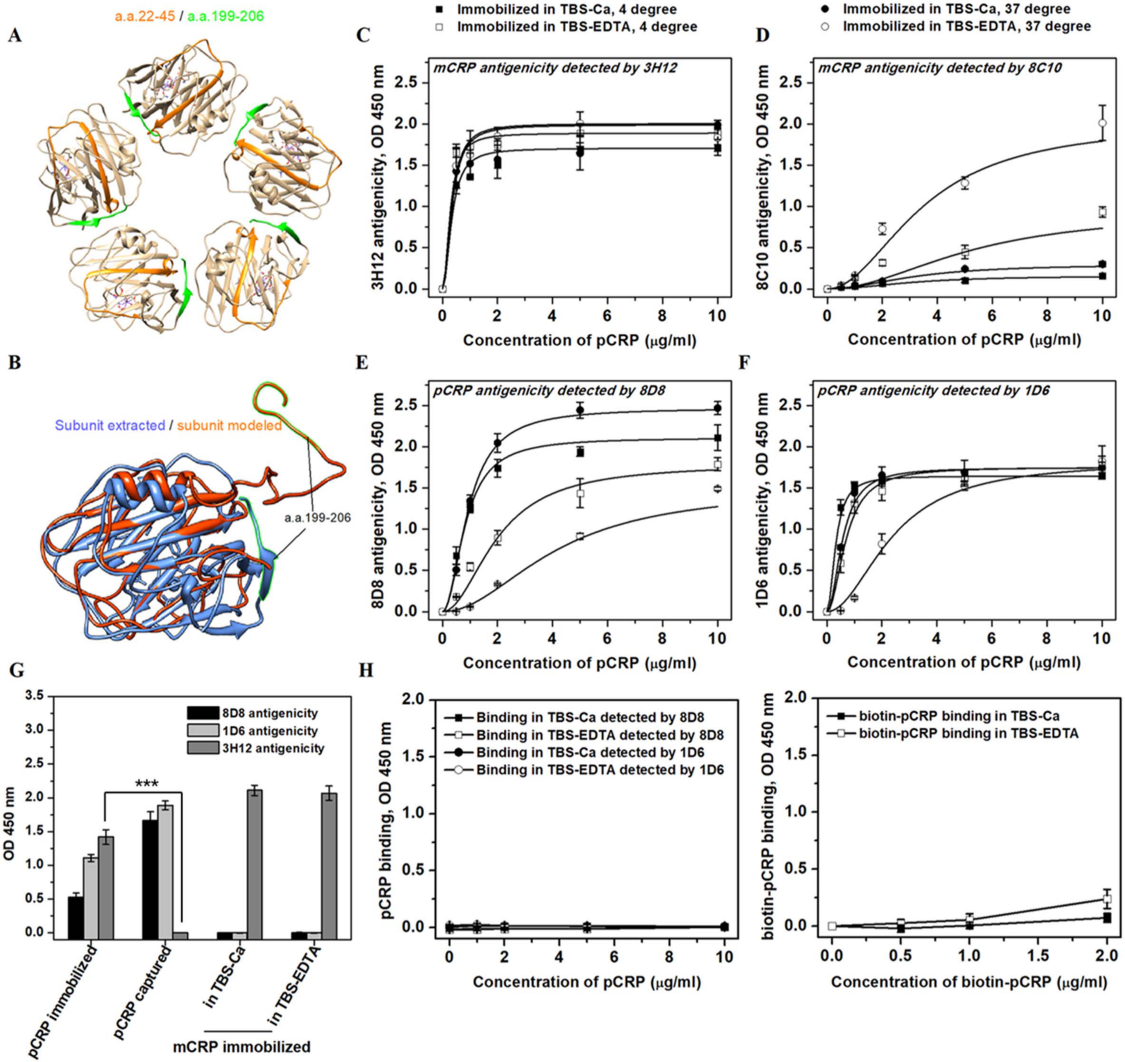
Surface-immobilization of pCRP induced the formation of an intermediate with dual antigenicity. (A) a.a.22-45 (recognized by 8C10; orange) is mostly packed inside the subunit in pCRP (PDB ID: 1B09), while a.a.199-206 (recognized by 3H12; green) lies at the subunit interface of the pentamer. (B) The subunit structures extracted from the crystal structure of pCRP (medium blue; PDB ID: 1B09) or de novo modeled by I-TASSER (orange red; C-Score = -0.78) were superposed. For I-TASSER modeling, templates with a homology over 25% were excluded. a.a.199-206 segment lies on the subunit interface of pCRP and packs closely against the subunit body. By contrast, this segment extrudes from the subunit body in the modeled structure, which is why 3H12 can report the pentamer disassembly. (C-F) Indicated concentrations of pCRP in TBS-Ca or TBS-EDTA (pH 7.4) were added into Nunc MaxiSorp microtiter wells and incubated overnight at 4°C or for 1 h at 37°C. The antigenicity expression of the immobilized protein was assayed with mAbs 3H12 (C), 8C10 (D), 8D8 (E) or 1D6 (F). Immobilized pCRP showed dual antigenicity of both pCRP (8D8 and 1D6) and mCRP (3H12 and 8C10). (G) 5 μg/ml pCRP or mCRP was immobilized in bicarbonate coating buffer (pH 9.6), TBS-Ca or TBS-EDTA (pH 7.4) at 4°C overnight. Alternatively, 2 μg/ml pCRP was captured by immobilized polyclonal sheep anti-human CRP antibody for 1 h at 37°C in TBS-Ca (pH 7.4). The antigenicity of the immobilized or bound antigens were determined by 8D8, 1D6 or 3H12. When mCRP was immobilized, only mCRP antigenicity (3H12) could be detected. By contrast, pCRP bound to immobilized pAb showed only pCRP antigenicity (8D8 and 1D6). However, directly immobilized pCRP showed significant 3H12 antigenicity (***p < 0.001). (H) Binding of unlabeled (the left panel) or biotin-labeled pCRP (the right panel) to mCRP (5 μg/ml) immobilized in TBS-Ca (pH 7.4) overnight at 4°C. The binding status was detected by mAbs and HRP-avidin, respectively. There was no binding of pCRP to the immobilized mCRP, excluding the possibility that the dual antigenicity was contributed by mCRP and survived pCRP. Data were obtained from at least three independent experiments and represented as mean ± SEM. For C-F, values underwent a nonlinear curve fit with OriginPro 8 software, during which the category was set as “Growth/Sigmoidal” and the function was set as “Hill1”.

Conformational conversion of pCRP to mCRP was shown critical to the function of CRP *in vivo*[6, 9, 10]. The membrane-induced dissociation of pCRP is suggestive and highlights the interplay between CRP functions and conformation[8]. However, the preparation of the membrane-induced conversion intermediate is complex and time-consuming. In order to make the evaluation easier, we tried here to simplify the process generating CRP intermediate. Specifically, two types of microtiter wells with hydrophilic and hydrophobic surfaces were used in the present study to mimic multivalent ligands and damaged cell membranes, respectively. We immobilized pCRP onto the surfaces and detect whether similar results of conformation conversion could be obtained as membrane-inducing. For this purpose, we first characterized the exact conformation of pCRP upon surface immobilization by using highly selective mAbs, i.e., 3H12, 8C10, 8D8, 1D6[8, 17–20]. Briefly, 8D8 and 1D6 detect the spatial conformation of native CRP, while 3H12 and 8C10 recognize linear epitopes exposed only in mCRP. Specifically, 8D8 and 1D6 recognize the native subunit conformations that are related to C1q- and calcium-binding[17], respectively. The C1q-recognizing face and calcium-binding face are on the opposite sides of CRP structure. By contrast, the linear epitope recognized by 3H12 (a.a.199-206) largely lies on the subunit contact interface of pCRP, only when the pentamer disassembly happens will its antigenicity express (Fig 1A and 1B); while the linear epitope of 8C10 (a.a.22-45) is mostly packed inside the subunit, which will be exposed when the native structure of dissociated subunit is further changed (Fig 1A). Hence, 3H12 antigenicity is a sensitive index for pCRP disassembly while 8C10 antigenicity expression reflects further and extensive remodeling of dissociated subunit structure.

As shown in Fig 1C and 1D, immobilization of pCRP onto hydrophilic surfaces (Nunc Maxisorp or Costar High Binding) in TBS-EDTA (pH 7.4) at 37°C resulted in a prominent expression of 3H12 and 8C10 antigenicity, indicating that pCRP was disassembled and converted to exposing mCRP epitopes. However, the conversion appeared to be partial as there was still significant binding of 8D8 and 1D6 left (Fig 1E and 1F). What interested us here was that immobilization of pCRP in calcium presence still generated dual antigenicity of both pCRP and mCRP. The calcium inclusion markedly increased the signal of 8D8 and 1D6 as expected; however, it was surprising that even calcium presence failed to prevent 3H12 and 8C10 antigenicity expression here (especially the former; Fig 1C–1F), indicating potential occurrence of pentamer disassembly. As controls, pCRP captured by the immobilized polyclonal anti-CRP exhibited only 8D8 and 1D6 antigenicity; while immobilized mCRP only expressed 3H12 and 8C10 antigenicity (Fig 1G). Comparable results were obtained using hydrophobic microtiter wells from two providers (Nunc PolySorp and JET High Binding) except for the more pronounced expression of mCRP antigenicity (S1 Fig).

There are two possibilities allowing dual antigenicity to arise, i.e., dual antigenicity here represents the mixture of intact pCRP and converted mCRP, or it represents CRP conformational intermediate with characters of both pCRP and mCRP.

To distinguish between these two possibilities, we next asked firstly whether the dual antigenicity represented mixture of intact pCRP and converted mCRP. Considering the surface property of the microtiter wells and the long incubation time (up to 18 h), it is unlikely that part of the immobilized pCRP were converted to mCRP while others were resistant to the conversion. Alternatively, the immobilized protein may completely dissociate into mCRP, and the pCRP antigenicity was contributed by the post-binding of the residue pCRP in the solution-phase as mCRP has been suggested to form aggregates [21–23]. In order to verify this, we checked the binding of unlabeled and biotin-labeled pCRP to the surface-immobilized mCRP in TBS-Ca. However, no binding could be observed (Fig 1H). Results above thus implicated that the current dual antigenicity did not result from the mixture of completely dissociated mCRP and surviving pCRP. Instead, it indeed represented conformational intermediate with characters of both pCRP and mCRP.

Therefore, we note with pleasure that surface immobilization of pCRP has the similar effect as membrane-inducing to generate a CRP intermediate, which is probably dissociated subunit remaining its near-native conformation. Here we term this intermediate CRP_i_.

**Fig 2:**
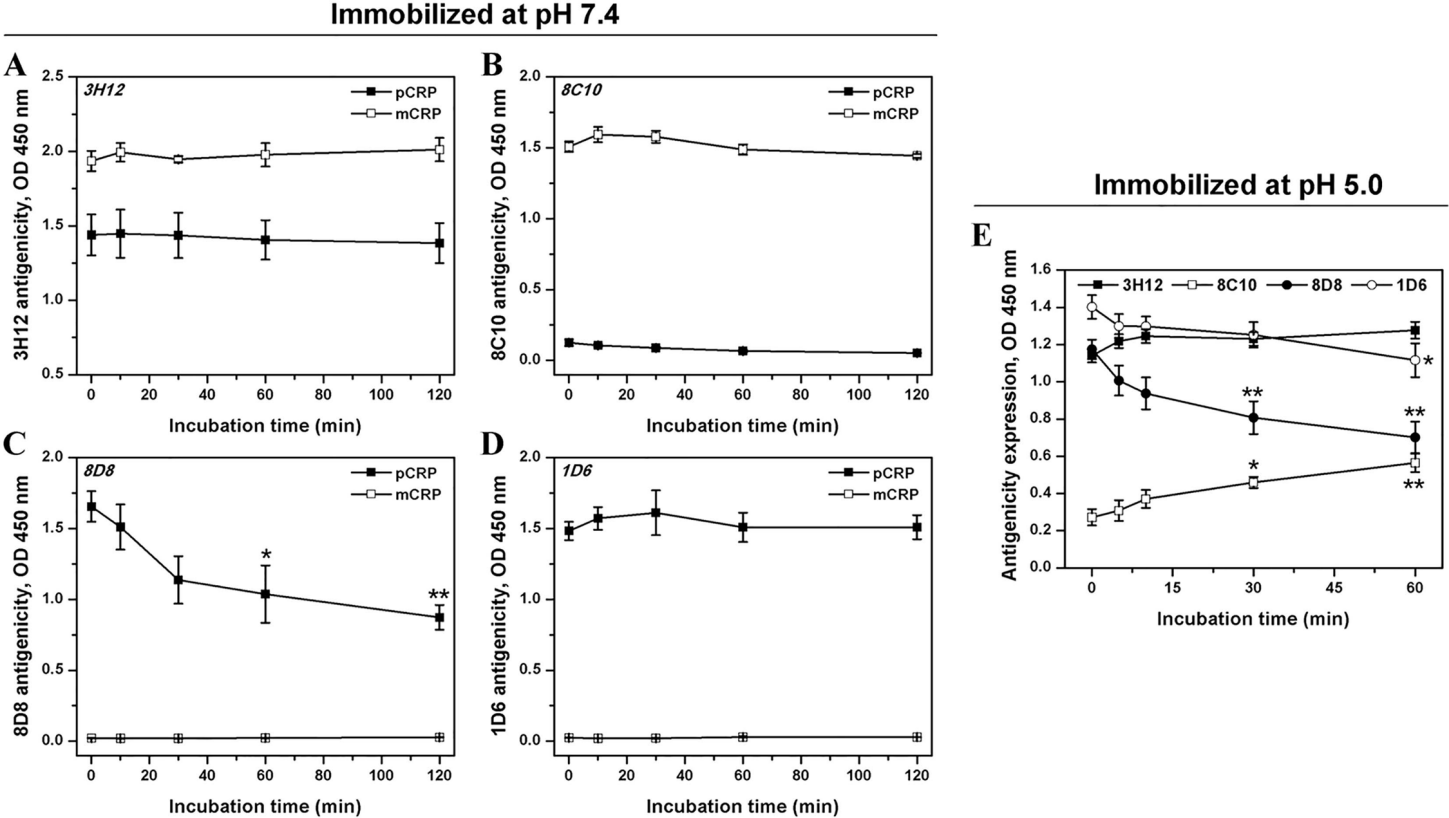
The formation of surface-induced intermediate precedes the loss of native subunit conformation. pCRP or mCRP was immobilized onto hydrophilic microtiter wells (Nunc MaxiSorp) for 3 min in TBS-Ca (pH 7.4) at 37°C. After brief washes, the immobilized proteins were further incubated for 0–2 h followed by antigenicity detection with 3H12 (A), 8C10 (B), 8D8 (C) or 1D6 (D). To increase the time resolution, the 1-h BSA blocking step before mAb addition was omitted in this series of experiment with only marginal increase in the background signal. Surface-immobilization prominently dissociated pCRP, as was reported by the increased 3H12 expression. However, the dissociated subunit still retained near-native conformation. (indicated by minimal 8C10 expression and unchanged 1D6 expression). As the control, the antigenicity expression of immobilized mCRP did not show time-dependent changes. (E) pCRP was immobilized onto hydrophilic microtiter wells (Costar High Binding) for 3 min in TBS-Ca (pH 5.0) at 37°C followed by 0–2 h incubation and detection of antigenicity expression. Lowering pH had little effect on the process of pentamer disassembly (changes of 3H12 and 8D8 showed the same tendency as above) but resulted in continuous structure remodeling of the structure of the dissociated subunit (8C10 rise and 1D6 decrease). Data were obtained from at least three independent experiments and represented as mean ± SEM. *p < 0.05, **p < 0.01 against the corresponding OD values at the beginning time point (0 minutes of incubation).

To further dissect the progress of the hybrid formation, additional experiments were carried out. Firstly, we asked how rapid this conformational conversion happened. The time course of the immobilization-induced conformational rearrangements was thus examined by using the conformation specific mAbs (8D8, 1D6, 3H12 and 8C10) above. With these mAbs, we found that the 3H12 antigenicity almost approached its maximal expression immediately after protein immobilization for 3 minutes, indicating an instant and complete disruption of the pentameric assembly of pCRP (Fig 2A). Conversely, the 8C10 antigenicity kept minimal expression throughout the phases, suggesting negligible further changes in the native structure of the dissociated subunits (Fig 2B). By contrast, nearly half of the 8D8 antigenicity expression which reporting the native CRP conformation was lost within 2 hours after immobilization (Fig 2C), also indicating the immobilization-induced disassembly of pCRP. However, there was little if any concomitant alteration in the 1D6 signal (Fig 2D). Here it is interesting to see the different results of 8D8 and 1D6. The two mAbs both report the native structure of CRP. However, the rationales are different. 8D8 recognizes the native subunit conformations of CRP that are related to C1q-binding. The C1q-binding site is supposed to be formed by two neighboring subunits in pCRP[24], thus 8D8 probably indirectly detects the intactness of the pentameric assembly by measuring the distance between subunits. By contrast, 1D6 recognizes native CRP in a calcium-dependent manner[17]. The calcium-binding site of CRP locates in the opposed face of C1q binding and mainly involves a series of spatially adjacent residues[12, 25], and only when the subunit conformation remains native will these residues be correctly positioned. Thus, the unchanged 1D6 signal here probably reflected that the dissociated subunit remained its native conformation. Remarkably, the relatively slower change in 8D8 antigenicity comparing with that of 3H12 likely represents the lag between the assembly interfaces disruption of pentamer and the epitope remodeling of dissociated subunits. As the control, no change in the antigenicity expression of immobilized mCRP could be observed with time (Fig 2). These results together further demonstrate that the hybrid conformation formed upon surface-immobilization of pCRP indeed represents dissociated subunit with near native structure, and the generation of CRP_i_ by immobilization-inducing is rapid (less than 3 minutes).

Comparing with the immediate rise of 3H12 antigenicity expression and the relatively slower loss of 8D8 signal, the strong 1D6 and minimal 8C10 antigenicity expression of hybrid remained unchanged over time (Fig 2B and 2D). These results lead to the speculation that even if in the presence of calcium, multi-point attachment of pCRP can efficiently dissociate pCRP, but it is insufficient on its own to further unfold the dissociated, near-native subunit into the mCRP conformation. Thus, a relatively stable intermediate of CRP was generated here. Furthermore, considering the extracellular acidification frequently occurs in inflammatory loci where CRP ligands are enriched[26–29], we examined the modulation of low pH on the formation and stability of the hybrid. We found that pH 5.0 had little effects on the pentamer disassembly process, as the time-dependent changes in 3H12 and 8D8 antigenicity expression were comparable to that obtained under neutral pH (Fig 2A and 2C). While it should be noted that, a gradual remodeling of the dissociated subunit structure was evidenced by the time-dependent rise and decline in antigenicity expression of 8C10 and 1D6, respectively (Fig 2E). Likewise, the combination of multi-point attachment and hydrophobicity by immobilization of pCRP onto hydrophobic surfaces, mirroring the interaction of pCRP with damaged membranes, resulted in even more rapid and pronounced rearrangements of the subunit structure (S2 Fig). The above results thus reveal that upon surface-binding, the pentamer dissociation and the native conformation loss of dissociated subunit can be separate events that occur in a stepwise manner, depicting a set of processes in the conformational conversion of CRP. Specifically, the conversion follows an order of “pCRP→CRP_i_→mCRP".

### Surface-induced CRP intermediate exhibited modified biological functions

**Fig 3:**
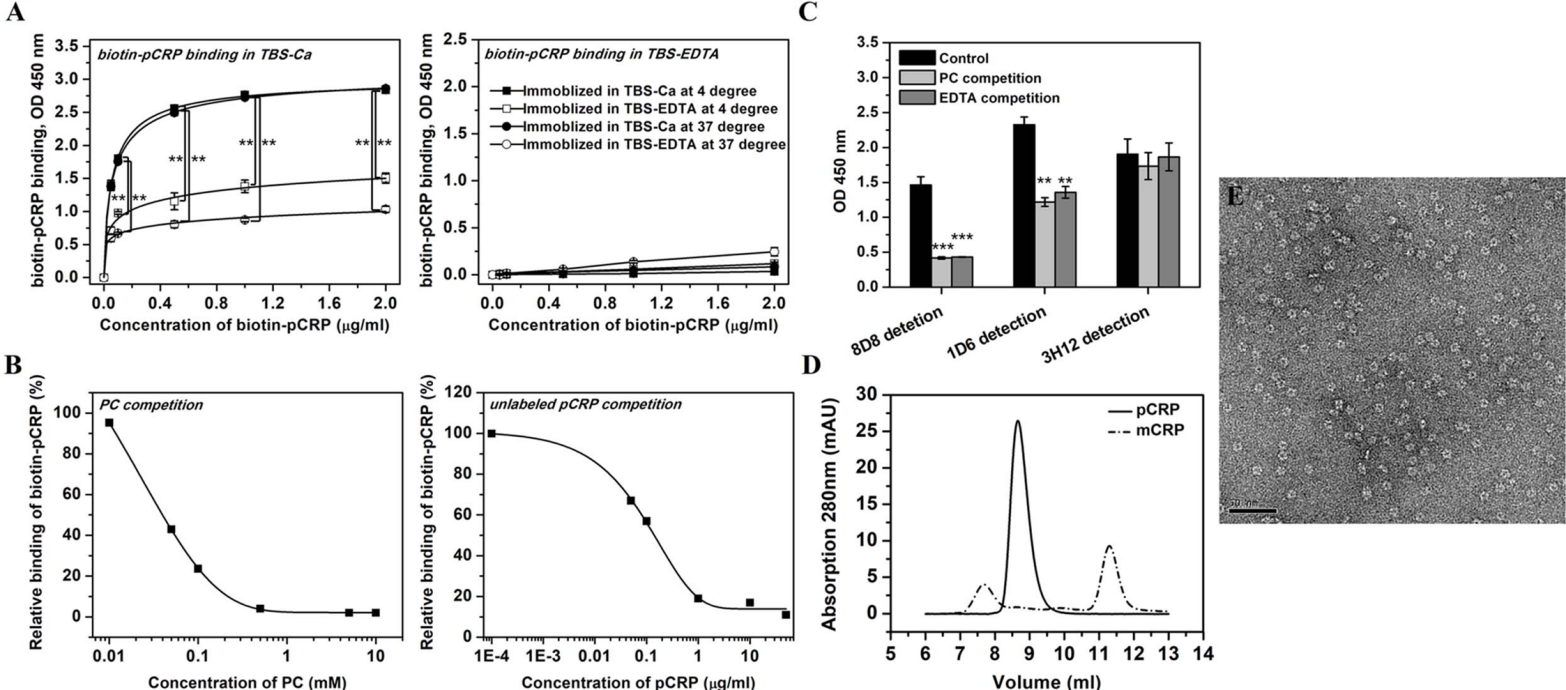
The intermediate formed on hydrophobic surfaces binds the solution-phase pCRP in a calcium-dependent manner with high affinity. (A) Binding of biotin-labeled pCRP to pCRP (5 μg/ml) immobilized in TBS-Ca (pH 7.4) overnight at 4°C. Biotin-labeled pCRP bound immobilized pCRP in a calcium-dependent fashion. **p < 0.01. This interaction affinity was much higher than the previously determined K_d_ for the homo-complex formation of pCRP (~1.5 nM vs. ~20 μM). (B) 5 μg/ml pCRP was immobilized in TBS-Ca (pH 7.4) overnight at 4°C. After incubation with PC (the left panel) or unlabeled pCRP (the right panel) at the indicated concentrations for 30 min, 0.2 μg/ml biotin-labeled pCRP was added for binding detection by HRP-avidin. The binding of biotin-labeled pCRP could be abrogated by PC and unlabeled pCRP. The results of one representative experiment were shown. (C) 5 μg/ml pCRP was immobilized in TBS-Ca (pH 7.4) overnight at 4°C followed by incubation with TBS-EDTA, 1% BSA or 2 mM PC in TBS-Ca (pH 7.4), 1% BSA for 1 h. The PC or EDTA treatments both significantly eliminated the post-binding of the solution-phase pCRP (**p < 0.01, ***p < 0.001 against control). However, the immobilized antigen still showed significant dual antigenicity as detected by 8D8, 1D6 and 3H12 mAbs. (D) Homogeneity analyzed by size-exclusion chromatography of pCRP and mCRP in solution phase. 120 μl of pCRP or mCRP at concentration of 0.1 mg/ml were injected and eluted from a Superdex 75 column attached to an AKTA purifier at a flow rate of 0.75 ml/min, respectively, and absorption values at 280nm were recorded. pCRP showed no heterogeneity while mCRP exhibited a certain amount of aggregations (E) Visualization of pCRP in TBS by electron-microscopy(EM). Scale bar = 50 nm. No apparent self-association of CRP like decamer was observed. Data were obtained from at least three independent experiments and represented as mean ± SEM unless otherwise stated. For A and B, values underwent a nonlinear curve fit with OriginPro 8 software, during which the category was set as “Growth/Sigmoidal” and the function was set as “Hill1”.

Then we asked whether this surface-induced CRP_i_ involved corresponding functional alterations. We first showed that compared with the immobilized mCRP (Fig 1H), pCRP immobilized onto hydrophobic surfaces possessed a high affinity (with a K_d_ value of about 1.5 nM) with the biotin-labeled pCRP in solution phase (Fig 3A, the left panel), and the binding was eliminated in TBS-EDTA (Fig 3A, the right panel), indicating in a calcium-dependent manner. Moreover, when the hybrid on the surfaces was pre-incubated with PC or unlabeled pCRP, the post-binding of biotin-labeled pCRP was abrogated (Fig 3B). In addition, despite that PC or EDTA treatments eliminated the post-binding of the solution-phase pCRP, the immobilized antigen still showed significant dual antigenicity as detected by 8D8, 1D6 and 3H12 mAbs (Fig 3C). Therefore, these results demonstrate a calcium-dependent binding of solution-phase pCRP to the surface-induced intermediate with a high affinity. It should be noted herein that the previously determined K_d_ for the homo-complex formation of pCRP is about 20μM[30], which is far below the present affinity. Indeed, the homogeneity of pCRP in solution phase under mild condition was further established by size-exclusion chromatography (SEC)[31] (Fig 3D) and electron microscope (EM)[32–34] (Fig 3E). SEC analysis of pCRP showed a single peak, and no apparent self-association of CRP like decamer was observed by EM. Considering the potential effects of CRP self-association on its functions *in vivo*[30, 35], we thus speculate here that the intermediate, which indicating conformational conversion of CRP, possesses altered biological activities and may play essential roles in the inflammation regulation of CRP.

**Fig 4:**
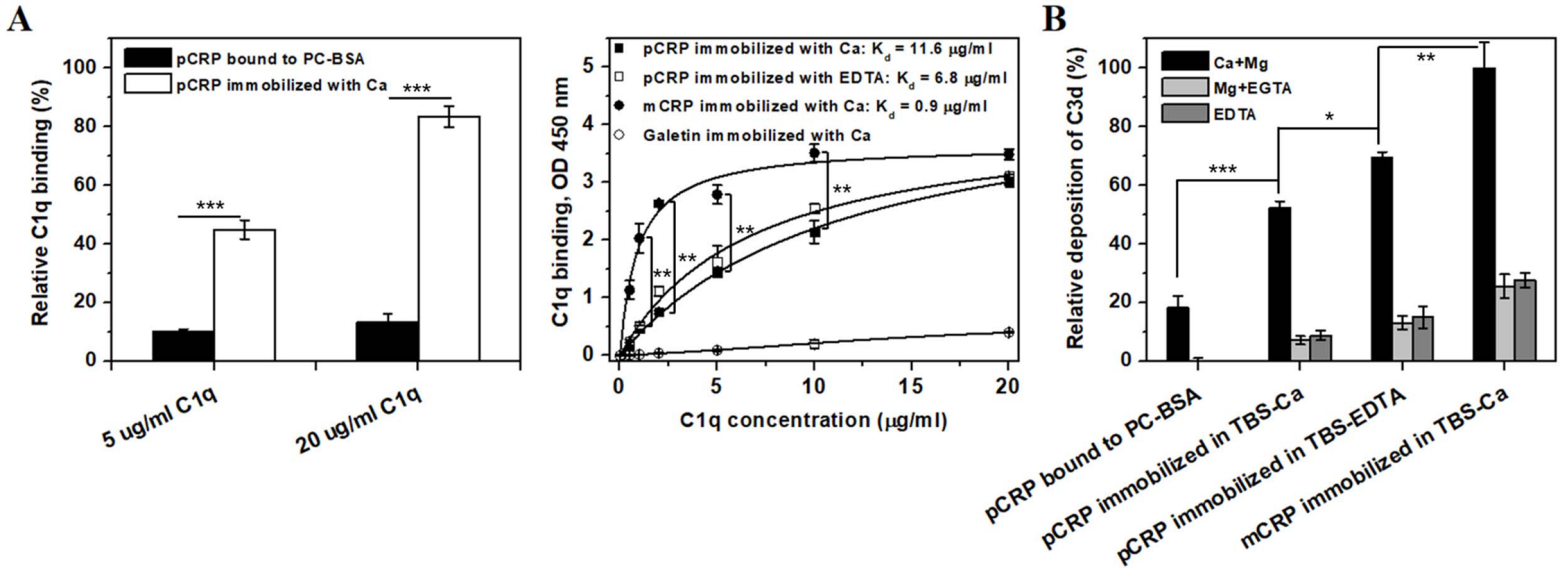
The surface-induced intermediate exhibited enhanced complement interaction capacity. 5 μg/ml pCRP or mCRP was immobilized in TBS-Ca or TBS-EDTA (pH 7.4) at 4°C overnight. Alternatively, 50 μg/ml pCRP was bound to immobilized PC-BSA overnight at 4°C in TBS-Ca (pH 7.4). The amount of pCRP bound to PC-BSA or immobilized onto surfaces was evaluated by a poly-clonal anti-CRP antibody and corrected when required. (A) C1q at the indicated concentrations was added to immobilized or bound proteins for 1 h followed by detection with C1q antibody. Directly immobilized pCRP exhibited three to five-fold stronger capacity than captured pCRP to bind C1q (***p < 0.001), and immobilized mCRP exhibited the highest C1q binding affinity of all (**p < 0.01: immobilized pCRP *vs* immobilized mCRP). (B) Wells were incubated with 5% normal human serum (NHS) diluted with VBS, 1% gelatin containing 0.15 mM Ca+0.5 mM Mg, 5 mM EGTA+0.5 mM Mg, or 5 mM EDTA, for 1 h at room temperature. The serums have been adsorbed with PC-BSA to eliminate trace level of CRP and anti-PC antibodies. The activation of complement was evaluated by C3d deposition. The bound and immobilized proteins all interacted with C1q and activated solely the classical complement pathway with the capacities in an increasing order of pCRP bound to immobilized PC-BSA, pCRP immobilized in TBS-Ca, pCRP immobilized in TBS-EDTA, immobilized mCRP. *p < 0.05; **p < 0.01; ***p < 0.001. All data were obtained from at least three independent experiments and represented as mean ± SEM. For A right, values underwent a nonlinear curve fit with OriginPro 8 software, during which the category was set as “Growth/Sigmoidal” and the function was set as “Hill1”.

Furthermore, we compared C1q binding capacity and complement activation of PC-BSA captured pCRP, immobilized pCRP and mCRP[8, 24] (Fig 4). Upon pCRP was binding to immobilized PC-BSA[8] or directly immobilized onto hydrophilic surfaces with calcium (Figs 1 and 2), the native subunit structure was barely altered; however, the intact pentameric assembly was kept only in the former case. As such, directly immobilized pCRP exhibited three to five-fold stronger capacity than captured pCRP to bind C1q (Fig 4A, the left panel). Considering the difference between the disassembly of surface-induced pCRP and intact assembly of PC-BSA-captured pCRP, we thus suspect that dissociation of the pentamer significantly enhanced the binding capacity of C1q to pCRP probably by overcoming the size limitation that allows only one of the five binding sites in pCRP to be bound by C1q[8]. Moreover, Immobilization of pCRP in TBS-EDTA resulted in about 1.7-fold increases in C1q binding capacity compared with immobilization when calcium was present (Fig 4A, the right panel), which was associated with the further structure remodeling of dissociated subunits as indicated by the upregulated 8C10 antigenicity expression (Fig 1D). As was expected, immobilized mCRP which may possess minimal size limitation, exhibited highest affinity with C1q (Fig 4A, the right panel). In addition, the activation of complement was further evaluated by C3d deposition[8]. We use Ca+Mg, EDTA+Mg and EDTA to control conditions involving classical/alternative, alternative and no-specific complement pathway activation, respectively[8]. The PC-BSA captured and directly immobilized proteins all activated solely the classical activation pathway, and the capacities were in an ascending order of PC-BSA-captured pCRP, immobilized pCRP in TBS-Ca, immobilized pCRP in TBS-EDTA, immobilized mCRP (Fig 4B), which agrees with the order of C1q-binding. It should be noted that mCRP showed most efficient C1q-binding and C3d deposition capacity, due to its completely lost of native subunit conformation (Figs 1 and 2). These results indicate that the surface-induced intermediate of CRP indeed exhibits modified biological functions and the bioactivities of CRP are fine-tuned by the subunit conformation.

## Discussion

Conversion of pCRP to mCRP is essential to the biological functions of CRP[8, 15, 16]. In contrast to the activated platelets or membrane-induced conformation conversion of CRP[8, 15], the present study actually has proposed another circumstance for pCRP dissociation occurring, which agrees well with the previous study and propose a simpler way to generate the CRP intermediate.

pCRP captured by the immobilized PC-BSA was shown to express single antigenicity here. Considering the different features of immobilized PC-BSA, cell membrane, liposomes and surfaces, we speculate that multipoint attachment may play a critical role in facilitating the conformational change of pCRP. But it should be noted that although multipoint attachment can dissociate pCRP efficiently in the presence of calcium, it was insufficient on its own to unfold the dissociated, native subunit to mCRP conformation further. Moreover, we showed that lower pH may facilitate the post-stage of the “pCRP→mCRP" conversion, indicating a critical role of extracellular acidification in inflammatory loci in pCRP dissociation and mCRP generation *in vivo*.

The surface-induced intermediate here exhibited highly enhanced affinity to the solution-phase pCRP. Considering that the self-association of pCRP was shown to affect its functions[30, 35]. We thus speculate that the membrane-bound CRP possesses modified potentials to interact with the free pCRP in inflammatory sites and performs corresponding bioactivities, where further studies are needed.

pCRP with no ligand binding shows only very weak interaction with C1q because of the size limitation of binding [8, 24]. By contrast, mCRP has been shown to regulate complement activation in a more effective way[7, 8, 10]. Indeed, the order of complement interaction capacity here was shown to be immobilized mCRP > immobilized pCRP > pCRP captured by immobilized PC-BSA. The enhanced C1q activation of immobilized pCRP comparing to the captured pCRP further suggests that the intermediate formed on the surfaces probably represents an intermediate during the transition of pCRP to mCRP, highlighting the correlation between the biological function and conformation of CRP.

In the present work, the immobilization-induced CRP_i_ remained binding on the surface for long incubation times. Usually, we believe that the binding of CRP_i_ to surface is relatively stable, and they may have function directly. However, we still cannot exclude the possibility small portions of CRP_i_ detaching from the surface occasionally. Recently, Lin Zhang *et*.*al* has revealed the existence of free mCRP in plasma[36], suggesting the detachment of membrane-dissociated CRP_i_ from the inflammatory loci.

Overall, we showed a surface-induced conformational conversion of CRP and identified a CRP intermediate with hybrid conformations and modified bioactivities. This simpler way to prepare CRP intermediate reveals the conversion details of pCRP to mCRP and highlights the relationship between biological function and conformation conversion of CRP.

## Supporting information

S1 Fig Immobilized pCRP exhibited dual antigenicity on hydrophobic surfaces. Indicated concentrations of pCRP were immobilized onto JET High Binding microtiter wells in TBS-Ca or TBS-EDTA (pH 7.4) overnight at 4°C or for 2 h at 37°C. The antigenicity expression of the immobilized protein was assayed with mAbs 3H12 (A), 8C10 (B), 8D8 (C) or 1D6 (D). 8D8 and 1D6 detect conformational epitopes unique to pCRP, whereas 3H12 and 8C10 recognize linear sequence epitopes exposed only in mCRP. Immobilized pCRP showed antigenicity of both pCRP and mCRP. (E) 5 μg/ml pCRP or mCRP was immobilized in bicarbonate coating buffer (pH 9.6), TBS-Ca or TBS-EDTA (pH 7.4) at 4°C overnight. Alternatively, 2 μg/ml pCRP was captured by immobilized polyclonal sheep anti-human CRP antibody for 1 h at 37°C in TBS-Ca. The antigenicity of the immobilized or bound antigens were determined by 8D8, 1D6 or 3H12. When pCRP was immobilized in coating buffer, or when mCRP was immobilized in TBS-Ca or TBS-EDTA, only mCRP antigenicity could be detected. By contrast, pCRP bound to immobilized pAb showed only pCRP antigenicity. Data were obtained from at least three independent experiments and represented as mean ± SEM. For A-D, values underwent a nonlinear curve fit with OriginPro 8 software, during which the category was set as “Growth/Sigmoidal” and the function was set as “Hill1”.
(TIF)

S2 Fig Pentamer disassembly precedes the loss of native subunit conformation upon immobilization onto hydrophobic surfaces. pCRP was immobilized onto hydrophobic microtiter wells (JET High Binding) for 5 min in TBS-Ca (pH 7.4) with or without 2 mM PC at room temperature. After brief washes, the immobilized pCRP was further incubated in TBS-Ca for the indicated times (0–60 min) followed by antigenicity detection with 8D8 (A), 1D6 (B), 3H12 (C) or 8C10 (D) (n = 4–6). To increase the time resolution, the 1-h BSA blocking step before mAb addition was omitted with only marginal increase in the background signal. The inclusion of PC was to minimize the possible interference from the solution-phase binding of pCRP (please see Fig 3). As on hydrophilic surfaces, binding to hydrophobic surfaces also resulted in an instant disruption of the pentameric assembly as indicated by the near maximal expression of 3H12 antigenicity and a quick drop of 8D8 signal. By contrast, the rearrangements in subunit conformation was more rapid and pronounced. Indeed, a significant higher 8C10 antigenicity expression could be detected immediately after immobilization followed by a quicker decline in the expression of 1D6 antigenicity. These suggest that the pentamer dissociation precedes changes in the subunit structure. For pCRP immobilized without PC, an additional 5-min wash with TBS-Ca, 2 mM PC was included before time-specific incubation, hence introducing a 5-min delay compared with pCRP immobilized with PC. This delay eliminated the early changes in the time-dependent curves without the delay, confirming the time resolution of our assay. Data were obtained from at least three independent experiments and represented as mean ± SEM.
(TIF)

## References

[1] Pepys MB, Hirschfield GM. C-reactive protein: a critical update. The Journal of clinical investigation. 2003;111(12):1805–12. doi: 10.1172/JCI18921 12813013 10.1172/JCI18921 PMC161431

[2] Du Clos TW. Pentraxins: structure, function, and role in inflammation. ISRN inflammation. 2013;2013:379040 doi: 10.1155/2013/379040 24167754 10.1155/2013/379040 PMC3791837

[3] Kresl JJ, Potempa LA, Anderson BE. Conversion of native oligomeric to a modified monomeric form of human C-reactive protein. Int J Biochem Cell Biol. 1998;30(12):1415–26. 9924810 10.1016/s1357-2725(98)00078-8

[4] Potempa LA, Siegel JN, Fedel BA, Potempa RT, Gewurz H. Expression, detection and assay of a neoantigen (Neo-CRP) associated with a free, human C-reactive protein subunit ☆. Molecular Immunology. 1987;24(5):531–41. 2443837 10.1016/0161-5890(87)90028-9

[5] Khreiss T, Jozsef L, Potempa LA, Filep JG. Conformational rearrangement in C-reactive protein is required for proinflammatory actions on human endothelial cells. Circulation. 2004;109(16):2016–22. doi: 10.1161/01.CIR.0000125527.41598.68 15051635 10.1161/01.CIR.0000125527.41598.68

[6] Wu Y, Potempa LA, El Kebir D, Filep JG. C-reactive protein and inflammation: conformational changes affect function. Biological chemistry. 2015;396(11):1181–97. doi: 10.1515/hsz-2015-0149 26040008 10.1515/hsz-2015-0149

[7] Molins B, Pena E, de la Torre R, Badimon L. Monomeric C-reactive protein is prothrombotic and dissociates from circulating pentameric C-reactive protein on adhered activated platelets under flow. Cardiovascular research. 2011;92(2):328–37. doi: 10.1093/cvr/cvr226 21859817 10.1093/cvr/cvr226

[8] Ji SR, Wu Y, Zhu L, Potempa LA, Sheng FL, Lu W, et al Cell membranes and liposomes dissociate C-reactive protein (CRP) to form a new, biologically active structural intermediate: mCRP(m). FASEB journal: official publication of the Federation of American Societies for Experimental Biology. 2007;21(1):284–94. 17116742 10.1096/fj.06-6722com

[9] Verma S, Szmitko PE, Yeh ET. C-reactive protein: structure affects function. Circulation. 2004;109(16):1914–7. doi: 10.1161/01.CIR.0000127085.32999.64 15117860 10.1161/01.CIR.0000127085.32999.64

[10] Khreiss T, Jozsef L, Hossain S, Chan JS, Potempa LA, Filep JG. Loss of pentameric symmetry of C-reactive protein is associated with delayed apoptosis of human neutrophils. The Journal of biological chemistry. 2002;277(43):40775–81. doi: 10.1074/jbc.M205378200 12198121 10.1074/jbc.M205378200

[11] Liu D, Cowburn D. Combining biophysical methods to analyze the disulfide bond in SH2 domain of C-terminal Src kinase. Biophysics Reports. 2016;2(1):33–43. doi: 10.1007/s41048-016-0025-4 27819029 10.1007/s41048-016-0025-4 PMC5071372

[12] Lv JM, Lü SQ, Liu ZP, Zhang J, Gao BX, Yao ZY, et al Conformational folding and disulfide bonding drive distinct stages of protein structure formation. Scientific Reports. 2018;8(1). 10.1038/s41598-018-20014-y PMC5784126 29367639

[13] Potempa LA, Maldonado BA, Laurent P, Zemel ES, Gewurz H. Antigenic, electrophoretic and binding alterations of human C-reactive protein modified selectively in the absence of calcium. Mol Immunol. 1983;20(11):1165–75. 6656768 10.1016/0161-5890(83)90140-2

[14] Thiele JR, Zeller J, Bannasch H, Stark GB, Peter K, Eisenhardt SU. Targeting C-Reactive Protein in Inflammatory Disease by Preventing Conformational Changes. Mediators Inflamm. 2015;2015:372432 doi: 10.1155/2015/372432 26089599 10.1155/2015/372432 PMC4451254

[15] Eisenhardt SU, Habersberger J, Murphy A, Chen YC, Woollard KJ, Bassler N, et al Dissociation of pentameric to monomeric C-reactive protein on activated platelets localizes inflammation to atherosclerotic plaques. Circ Res. 2009;105(2):128–37. doi: 10.1161/CIRCRESAHA.108.190611 19520972 10.1161/CIRCRESAHA.108.190611

[16] Thiele JR, Habersberger J, Braig D, Schmidt Y, Goerendt K, Maurer V, et al Dissociation of pentameric to monomeric C-reactive protein localizes and aggravates inflammation: in vivo proof of a powerful proinflammatory mechanism and a new anti-inflammatory strategy. Circulation. 2014;130(1):35–50. doi: 10.1161/CIRCULATIONAHA.113.007124 24982116 10.1161/CIRCULATIONAHA.113.007124

[17] Ying SC, Gewurz H, Kinoshita CM, Potempa LA, Siegel JN. Identification and partial characterization of multiple native and neoantigenic epitopes of human C-reactive protein by using monoclonal antibodies. J Immunol. 1989;143(1):221–8. 2471736

[18] Shi P, Li XX, Zhu W, Yang H, Dong C, Li XM. Immunohistochemical staining reveals C-reactive protein existing predominantly as altered conformation forms in inflammatory lesions. Acta Biol Hung. 2014;65(3):265–73. doi: 10.1556/ABiol.65.2014.3.3 25194730 10.1556/ABiol.65.2014.3.3

[19] Biro A, Rovo Z, Papp D, Cervenak L, Varga L, Fust G, et al Studies on the interactions between C-reactive protein and complement proteins. Immunology. 2007;121(1):40–50. doi: 10.1111/j.1365-2567.2007.02535.x 17244159 10.1111/j.1365-2567.2007.02535.x PMC2265924

[20] Udvarnoki K, Cervenak L, Uray K, Hudecz F, Kacskovics I, Spallek R, et al Antibodies against C-reactive protein cross-react with 60-kilodalton heat shock proteins. Clinical and vaccine immunology: CVI. 2007;14(4):335–41. doi: 10.1128/CVI.00155-06 17301219 10.1128/CVI.00155-06 PMC1865608

[21] Diehl EE, Radosevich JA, Potempa LA. Immunohistochemical localization of modified C-reactive protein antigen in normal vascular tissue. American Journal of the Medical Sciences. 2000;319(2):79–83. 10698090 10.1097/00000441-200002000-00002

[22] Molins B, Pena E, Vilahur G, Mendieta C, Slevin M, Badimon L. C-reactive protein isoforms differ in their effects on thrombus growth. Arteriosclerosis, thrombosis, and vascular biology. 2008;28(12):2239–46. doi: 10.1161/ATVBAHA.108.174359 18787187 10.1161/ATVBAHA.108.174359

[23] Slevin M, Krupinski J. A role for monomeric C-reactive protein in regulation of angiogenesis, endothelial cell inflammation and thrombus formation in cardiovascular/cerebrovascular disease? Histology & Histopathology. 2009;24(11):1473–8. 19760596 10.14670/HH-24.1473

[24] Agrawal A, Shrive AK, Greenhough TJ, Volanakis JE. Topology and Structure of the C1q-Binding Site on C-Reactive Protein. The Journal of Immunology. 2001;166(6):3998–4004. 11238646 10.4049/jimmunol.166.6.3998

[25] Thompson D, Pepys MB, Wood SP. The physiological structure of human C-reactive protein and its complex with phosphocholine. Structure. 1999;7(2):169–77. doi: 10.1016/S0969-2126(99)80023-9 10368284 10.1016/S0969-2126(99)80023-9

[26] Peisajovich A, Marnell L, Mold C, Clos TWD. C-reactive protein at the interface between innate immunity and inflammation. Expert Review of Clinical Immunology. 2008;4(3):379–90. doi: 10.1586/1744666X.4.3.379 20476927 10.1586/1744666X.4.3.379

[27] Hammond DJ JR., Singh SK, Thompson JA, Beeler BW, Rusinol AE, Pangburn MK, et al Identification of acidic pH-dependent ligands of pentameric C-reactive protein. J Biol Chem. 2010;285(46):36235–44. doi: 10.1074/jbc.M110.142026 20843812 10.1074/jbc.M110.142026 PMC2975246

[28] Park SY, Bae DJ, Kim MJ, Mei LP, Kim IS. Extracellular Low pH Modulates Phosphatidylserine-dependent Phagocytosis in Macrophages by Increasing Stabilin-1 Expression. Journal of Biological Chemistry. 2012;287(14):11261 doi: 10.1074/jbc.M111.310953 22334667 10.1074/jbc.M111.310953 PMC3322863

[29] Shrivastava AK, Singh HV, Raizada A, Singh SK. C-reactive protein, inflammation and coronary heart disease. Egyptian Heart Journal. 2015;67(2):89–97.

[30] Okemefuna AI, Stach LS, Buetas AJ, Gor J, Perkins SJ. C-reactive protein exists in an NaCl concentration-dependent pentamer-decamer equilibrium in physiological buffer. Journal of Biological Chemistry. 2010;285(2):1041–52. doi: 10.1074/jbc.M109.044495 19903811 10.1074/jbc.M109.044495 PMC2801231

[31] Kuhn E, Wu J, Karl J, Liao H, Zolg W, Guild B. Quantification of C-reactive protein in the serum of patients with rheumatoid arthritis using multiple reaction monitoring mass spectrometry and 13C-labeled peptide standards. Proteomics. 2004;4(4):1175–86. doi: 10.1002/pmic.200300670 15048997 10.1002/pmic.200300670

[32] Wang HW, Sui SF. Dissociation and subunit rearrangement of membrane-bound human C-reactive proteins. Biochemical and biophysical research communications. 2001;288(1):75–9. doi: 10.1006/bbrc.2001.5733 11594754 10.1006/bbrc.2001.5733

[33] Wang S, Li S, Ji G, Huang X, Sun F. Using integrated correlative cryo-light and electron microscopy to directly observe syntaphilin-immobilized neuronal mitochondriain situ. Biophysics Reports. 2017;3(1):8–16. doi: 10.1007/s41048-017-0035-x 28781997 10.1007/s41048-017-0035-x PMC5515996

[34] Liu Z, Zhang J. Exploring the inside details of virions by electron microscopy. Biophysics Reports. 2016;2(1):21–4. doi: 10.1007/s41048-016-0022-7 27819027 10.1007/s41048-016-0022-7 PMC5071365

[35] Pepys MB, Hirschfield GM, Tennent GA, Gallimore JR, Kahan MC, Bellotti V, et al Targeting C-reactive protein for the treatment of cardiovascular disease. Nature. 2006;440(7088):1217–21. doi: 10.1038/nature04672 16642000 10.1038/nature04672

[36] Zhang L, Li HY, Li W, Shen ZY, Wang YD, Ji SR, et al An ELISA Assay for Quantifying Monomeric C-Reactive Protein in Plasma. Frontiers in Immunology. 2018;9:511 doi: 10.3389/fimmu.2018.00511 29593741 10.3389/fimmu.2018.00511 PMC5857914

