# Supplementary figures and images for: In vitro generation and bioactivity evaluation of C-reactive protein intermediate
Source: PLoS One. 2018 May 31;13(5):e0198375. doi: 10.1371/journal.pone.0198375 (PMC5979025; doi:10.1371/journal.pone.0198375)

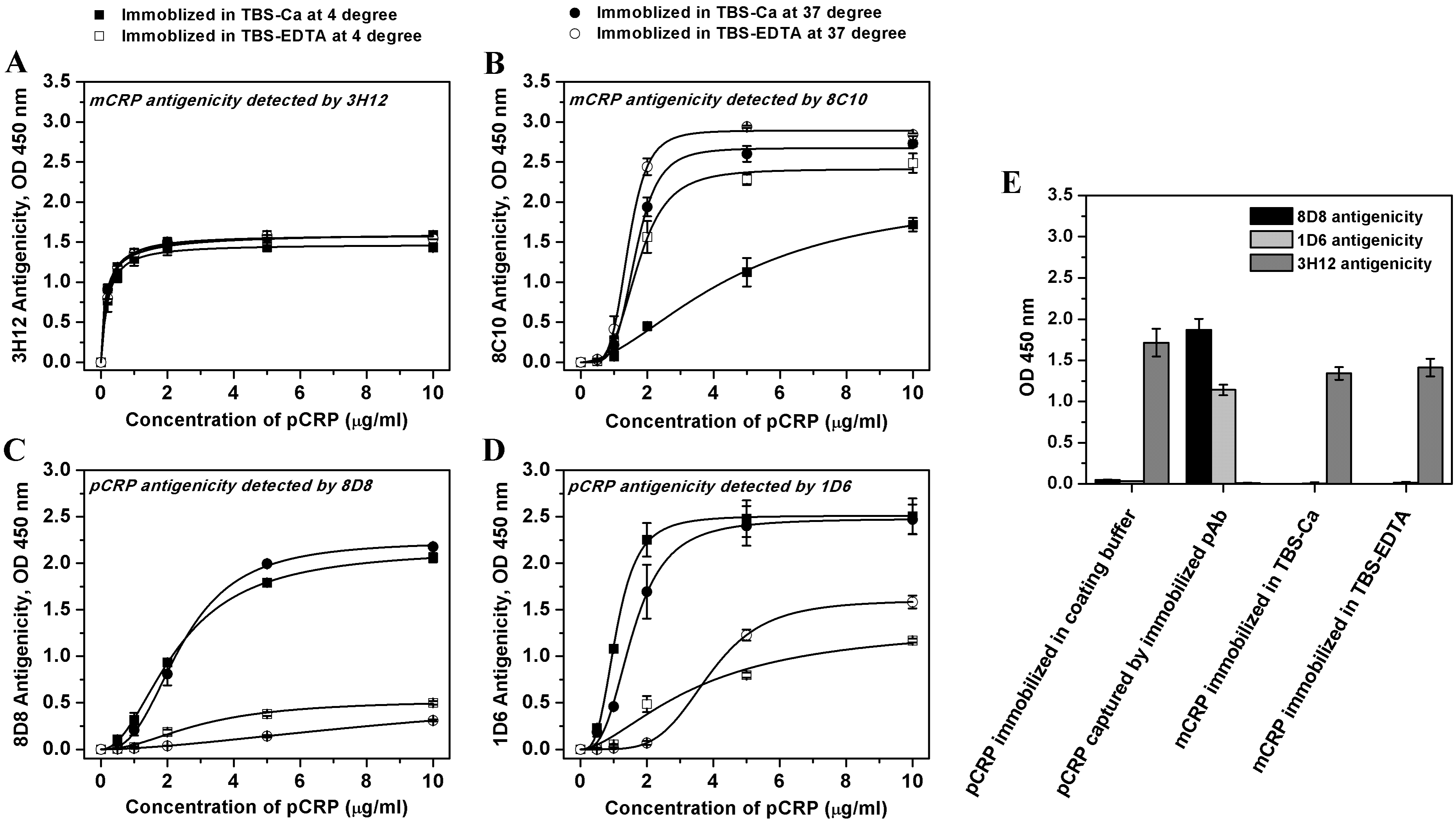

Supplement: S1 Fig — Indicated concentrations of pCRP were immobilized onto JET High Binding microtiter wells in TBS-Ca or TBS-EDTA (pH 7.4) overnight at 4°C or for 2 h at 37°C. The antigenicity expression of the immobilized protein was assayed with mAbs 3H12 (A), 8C10 (B), 8D8 (C) or 1D6 (D). 8D8 and 1D6 detect conformational epitopes unique to pCRP, whereas 3H12 and 8C10 recognize linear sequence epitopes exposed only in mCRP. Immobilized pCRP showed antigenicity of both pCRP and mCRP. (E) 5 μg/ml pCRP or mCRP was immobilized in bicarbonate coating buffer (pH 9.6), TBS-Ca or TBS-EDTA (pH 7.4) at 4°C overnight. Alternatively, 2 μg/ml pCRP was captured by immobilized polyclonal sheep anti-human CRP antibody for 1 h at 37°C in TBS-Ca. The antigenicity of the immobilized or bound antigens were determined by 8D8, 1D6 or 3H12. When pCRP was immobilized in coating buffer, or when mCRP was immobilized in TBS-Ca or TBS-EDTA, only mCRP antigenicity could be detected. By contrast, pCRP bound to immobilized pAb showed only pCRP antigenicity. Data were obtained from at least three independent experiments and represented as mean ± SEM. For A-D, values underwent a nonlinear curve fit with OriginPro 8 software, during which the category was set as “Growth/Sigmoidal” and the function was set as “Hill1”. (TIF) [file pone.0198375.s001.tif]

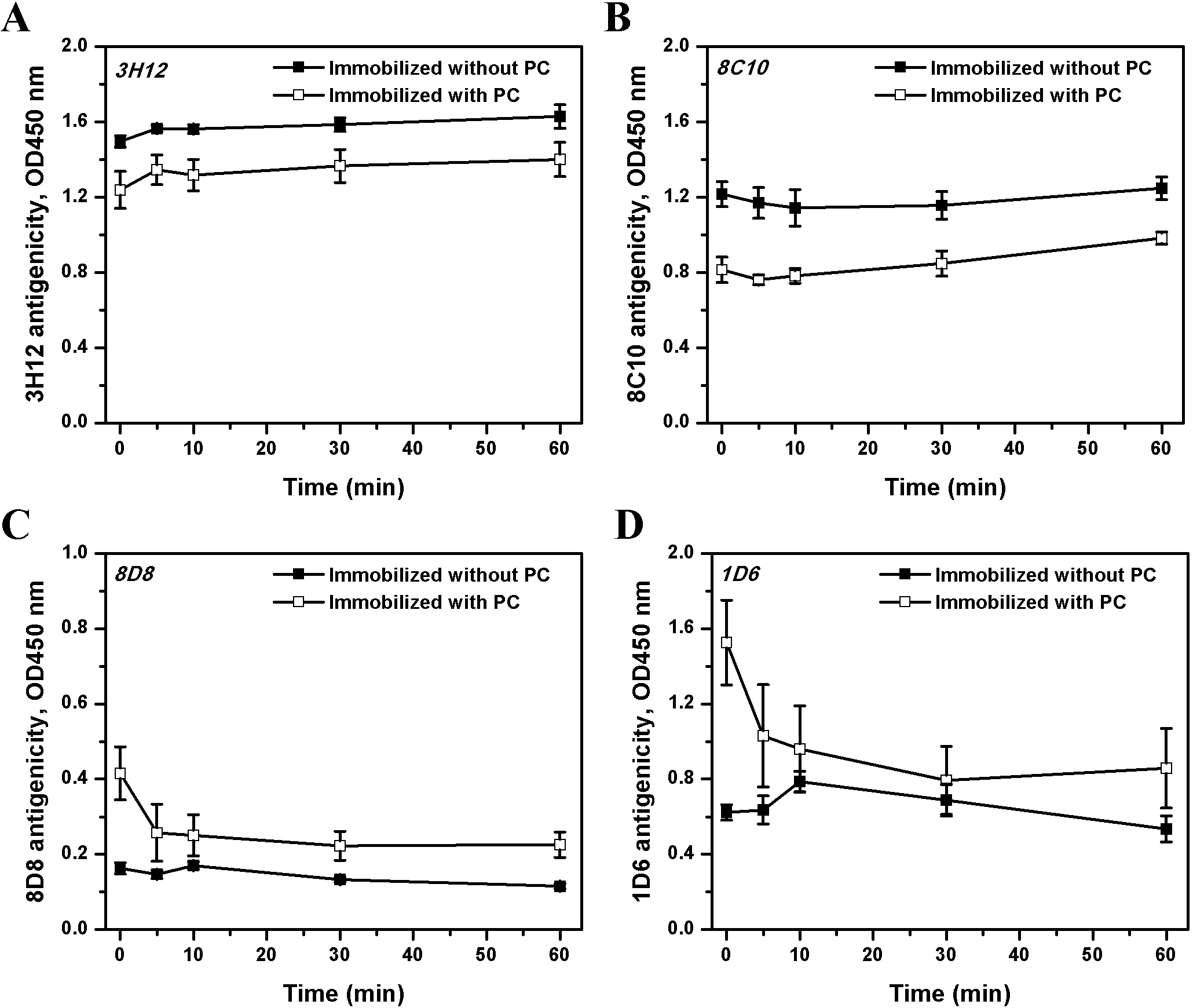

Supplement: S2 Fig — pCRP was immobilized onto hydrophobic microtiter wells (JET High Binding) for 5 min in TBS-Ca (pH 7.4) with or without 2 mM PC at room temperature. After brief washes, the immobilized pCRP was further incubated in TBS-Ca for the indicated times (0–60 min) followed by antigenicity detection with 8D8 (A), 1D6 (B), 3H12 (C) or 8C10 (D) (n = 4–6). To increase the time resolution, the 1-h BSA blocking step before mAb addition was omitted with only marginal increase in the background signal. The inclusion of PC was to minimize the possible interference from the solution-phase binding of pCRP (please see Fig 3). As on hydrophilic surfaces, binding to hydrophobic surfaces also resulted in an instant disruption of the pentameric assembly as indicated by the near maximal expression of 3H12 antigenicity and a quick drop of 8D8 signal. By contrast, the rearrangements in subunit conformation was more rapid and pronounced. Indeed, a significant higher 8C10 antigenicity expression could be detected immediately after immobilization followed by a quicker decline in the expression of 1D6 antigenicity. These suggest that the pentamer dissociation precedes changes in the subunit structure. For pCRP immobilized without PC, an additional 5-min wash with TBS-Ca, 2 mM PC was included before time-specific incubation, hence introducing a 5-min delay compared with pCRP immobilized with PC. This delay eliminated the early changes in the time-dependent curves without the delay, confirming the time resolution of our assay. Data were obtained from at least three independent experiments and represented as mean ± SEM. (TIF) [file pone.0198375.s002.tif]
